# Exploring the Prospects of Transcranial Electrical Stimulation (tES) as a Therapeutic Intervention for Post-Stroke Motor Recovery: A Narrative Review

**DOI:** 10.3390/brainsci14040322

**Published:** 2024-03-27

**Authors:** Hao Meng, Michael Houston, Yingchun Zhang, Sheng Li

**Affiliations:** 1 Department of Physical Medicine & Rehabilitation, McGovern Medical School, University of Texas Health Science Center at Houston, Houston, TX 77030, USA; 2 Department of Biomedical Engineering, University of Houston, Houston, TX 77204, USA; mjhousto@cougarnet.uh.edu; 3 Department of Biomedical Engineering, University of Miami, Coral Gables, FL 33146, USA; y.zhang@miami.edu; 4 TIRR Memorial Hermann Hospital, Houston, TX 77030, USA

**Keywords:** tES, tDCS, tACS, tRNS, stroke, motor recovery

## Abstract

Introduction: Stroke survivors often have motor impairments and related functional deficits. Transcranial Electrical Stimulation (tES) is a rapidly evolving field that offers a wide range of capabilities for modulating brain function, and it is safe and inexpensive. It has the potential for widespread use for post-stroke motor recovery. Transcranial Direct Current Stimulation (tDCS), Transcranial Alternating Current Stimulation (tACS), and Transcranial Random Noise Stimulation (tRNS) are three recognized tES techniques that have gained substantial attention in recent years but have different mechanisms of action. tDCS has been widely used in stroke motor rehabilitation, while applications of tACS and tRNS are very limited. The tDCS protocols could vary significantly, and outcomes are heterogeneous. Purpose: the current review attempted to explore the mechanisms underlying commonly employed tES techniques and evaluate their prospective advantages and challenges for their applications in motor recovery after stroke. Conclusion: tDCS could depolarize and hyperpolarize the potentials of cortical motor neurons, while tACS and tRNS could target specific brain rhythms and entrain neural networks. Despite the extensive use of tDCS, the complexity of neural networks calls for more sophisticated modifications like tACS and tRNS.

## 1. Introduction

Stroke results from damage to the central nervous system [1]. The typical symptoms caused by stroke can include motor deficits like muscle weakness, impaired coordination, and spasticity; cognitive impairments affecting memory, attention, and problem-solving; speech and language difficulties; and emotional disturbances, such as depression and anxiety [2]. Transcranial Electrical Stimulation (tES) is a rapidly developing field that has gained considerable attention for its potential in post-stroke motor recovery over the past two decades. This technique applies an electric field to the scalp surface to modulate brain activity. In fact, instead of using high-intensity stimulation current in the early efforts, contemporary tES applies a weak electric current (1~2 mA) to the scalp to modulate the cortical excitability [3]. tES can be classified into Transcranial Direct Current Stimulation (tDCS), Transcranial Alternating Current Stimulation (tACS), and Transcranial Random Noise Stimulation (tRNS) [4]. Compared to other Non-Invasive Brain Stimulation (NIBS) techniques like Transcranial Magnetic Stimulation (TMS), the popularity of tES arises from several factors. First, when applied within guidelines, it is non-invasive and relatively safe, with minimal side effects and risks. Second, its low cost and portability make it accessible for various research and therapeutic purposes. Finally, it offers ease of operation and customization, allowing researchers and clinicians to tailor its use to meet specific goals [5]. However, various tES techniques have limitations that challenge their clinical efficacy and the replication of research findings.

Recent reviews suggest that tDCS can modulate cortical excitability and potentially benefit motor recovery in stroke survivors [6,7,8,9]. However, when combined with physical therapy, several other reviews have indicated that tDCS might not consistently augment the effects [10,11,12,13]. While tDCS has been widely studied for stroke motor recovery, research on tACS in this field is still comparatively limited. Takeuchi and Izumi [14] reviewed the potential of tACS to enhance motor function and concluded that, although targeting brain oscillations with tACS shows promise for improving motor learning, further research is necessary to provide more conclusive evidence. The review also highlights the potential for a synergistic effect on motor learning when combining tACS with other neurorehabilitation methods. Yang et al. [15] also reviewed relevant tACS studies in stroke recovery, finding that tACS is linked to improvements in overall functional recovery, sensorimotor impairment, aphasia, and hemispatial neglect. Despite the common advantages, emerging tES methods employ distinct mechanisms to modulate cortical excitability, and the paradigms for applying tES are continually evolving. However, the efficacy of tES in stroke motor recovery presents challenges, and there is not yet a definitive conclusion regarding which technique could optimize the benefits of stroke neurorehabilitation. This review aims to explore the potential of tES for improving upper-limb motor recovery in stroke survivors. We will compare the mechanisms and neuromodulatory effects of tDCS, tACS, and tRNS in both healthy individuals and stroke patients. Additionally, we’ll analyze the advantages and disadvantages of each technique, suggesting future applications of tES in stroke motor recovery. The findings of this review will provide researchers with a deeper understanding of the mechanisms, paradigms, and potential future applications of tES in this important area.

## 2. Literature Search

We conducted a PubMed literature search using keywords “tDCS/tACS/tRNS”, “primary motor cortex”, “cortical excitability”, and “healthy/stroke” to identify relevant studies published between 2014 and 2024. We excluded studies that did not apply stimulation over the primary motor cortex. After applying these criteria, 79 studies were included: 54 investigating tDCS, 16 investigating tACS, and 9 investigating tRNS.

## 3. Stroke Upper Limb Motor Recovery

Muscle weakness or paralysis on one side of the body can severely impact upper limb function in stroke survivors. This impairment may present as difficulty performing simple movements or a complete inability to use the affected arm and hand. These motor deficits can significantly disrupt activities of daily living—dressing, feeding, and personal care—thus substantially diminishing the stroke survivor’s autonomy and life quality [16]. A previous review has indicated that approximately 80% of individuals post-stroke experience upper limb impairments early in the recovery process, with a minority achieving full functional restoration by six months [17]. Abnormal motor synergies can often be observed in the upper limb functions of stroke survivors, such as abnormal reaching movements characterized by shoulder abduction and elbow flexion instead of the normal shoulder flexion and elbow extension. Additionally, adaptations in reaching and grasping movements may occur due to sensory impairments [16]. In clinical settings, a well-accepted three-stage motor recovery framework has been proposed: flaccid, spastic, and recovered [18]. Recovery from stroke is a long journey; for some stroke survivors, it could last a lifetime. The success of stroke recovery requires collaboration among patients, doctors, therapists, and family members [19]. Current consensus indicates that rehabilitative interventions are most effective when they provide early, intensive, task-specific, and multisensory stimulation. Integrating both bottom-up and top-down processes is advantageous for promoting brain plasticity [20].

## 4. Transcranial Direct Current Stimulation (tDCS)

**Figure 1:**
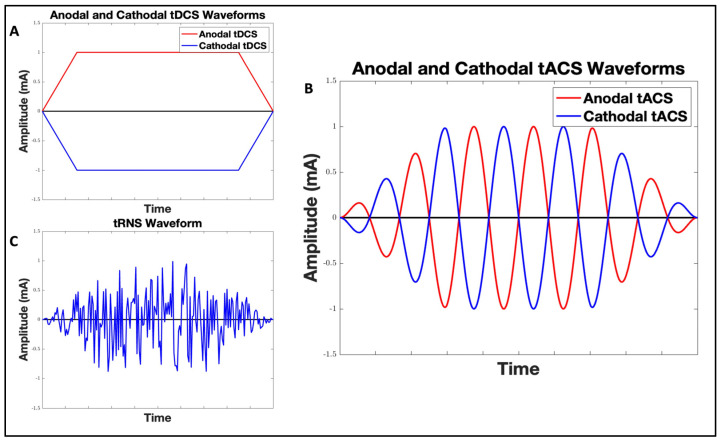
Demonstration of Transcranial Direct Current Stimulation (tDCS) (**A**), Transcranial Alternating Current Stimulation (tACS) (**B**), and Transcranial Random Noise Stimulation (tRNS) (**C**) waveforms. The red waveform represents the anodal current, and the blue waveform represents the cathodal current in tDCS and tACS. tDCS features a constant, flat waveform, while the waveform of tACS varies according to its frequency, and tRNS exhibits a waveform with a randomized frequency. In tDCS, the anode increases neural excitability while the cathode decreases it. However, tACS uses a sinusoidal waveform, meaning the current alternates between positive and negative values, minimizing the distinction between anodal and cathodal effects [29].

In the early 2000s, Nitsche and Paulus [21] proposed an approach to modulating cortical excitability by applying an anodal electrode that delivers constant current to the motor cortex and a cathodal electrode to the contralateral forehead (Figure 1A). They discovered that this specific electrode arrangement enhanced motor cortex excitability, attributed to the anodal stimulation depolarizing the motor neuron membrane, thereby potentiating action potentials. Conversely, cathodal stimulation results in the hyperpolarization of the membrane. This initial experiment with tDCS laid the groundwork for tES neuromodulation, leading studies to apply tDCS across various fields. Furthermore, at the molecular and cellular levels, the modulation associated with tDCS may be linked to activity in various neurotransmitter systems, including glutamatergic, GABAergic, dopaminergic, serotonergic, and cholinergic pathways [22]. In fact, the mechanism of tDCS can be interpreted in two parts: the acute effect (online effect) and the plastic effect (offline effect). In the acute phase, the action potential of the neuronal membrane is determined by afferent activity via electrical and chemical synapses and also by extra-synaptic substances, which activate specific ion channels and receptors [23,24,25]. On the other hand, neuroplasticity can also be observed following tDCS. Neuroplasticity refers to the brain’s ability to change its structure and function at the level of individual neurons or throughout entire neuronal networks [26]. When the membrane of glutamatergic synapses is depolarized or hyperpolarized, tDCS may increase or decrease the amount of calcium flow through the N-methyl-D-aspartate (NMDA) receptor and calcium channels. Depending on the changes in intraneuronal calcium levels, glutamatergic α-amino-3-hydroxy-5-methyl-4-isoxazolepropionic acid (AMPA) receptors can be inserted into or removed from the subsynaptic membrane, consequently improving or reducing synaptic connectivity [23,25,27]. Furthermore, changes in intracellular calcium levels can contribute to long-term potentiation (LTP) or long-term depression (LTD), which are further influenced by the intensity and varying protocols of tDCS [23,28].

Despite the diverse mechanisms proposed, tDCS has been extensively studied for its ability to modulate excitability in the motor cortex. Owing to its polarity feature, conventional tDCS applications involve placing the anodal electrode over the primary motor cortex (M1) and the cathodal electrode over the supraorbital region of the prefrontal cortex. In healthy subjects, applying the anodal electrode over M1 has shown modulation effects in numerous studies, employing a wide range of outcome measures. Notturno et al. [30] applied tDCS with an intensity of 1 mA for 20 min at the M1 area and observed an increase in cortical low alpha-band power and beta-band brain connectivity following anodal tDCS. Romero Lauro et al. [31] explored the broader effects of tDCS on cortical excitability, finding significant shifts in global excitability and increased cortical activity during and after anodal tDCS application. However, they also reported that both anodal and cathodal tDCS resulted in widespread changes in regional cerebral blood flow (CBF) compared to sham tDCS. Similarly, Jamil et al. [32] reported that anodal tDCS over M1 increased CBF, and more so at higher intensities. In addition, more recent studies have demonstrated increased Motor Evoked Potentials (MEPs) following a specific period of time and intensity of anodal tDCS stimulation over M1 [33,34,35,36,37,38].

In stroke survivors, damage to the motor cortex can lead to impaired motor function and muscle weakness. Given its ability to modulate cortical excitability, tDCS is increasingly applied in the rehabilitation of motor function in stroke survivors. In studies focusing on unilateral tDCS application with therapy, Allman et al. [39] combined anodal tDCS at the M1 ipsilesional site with the Graded Repetitive Arm Supplementary Program (GRASP) for 9 days. They reported significant improvements in the Action Research Arm Test (ARAT) and the Wolf Motor Function Test (WMFT) among the anodal tDCS group, along with increased cortical activity in the ipsilesional premotor and motor areas from fMRI and longer retention of benefits compared to the sham group. Halakoo et al. [40] evaluated the impact of anodal tDCS on spasticity and muscle activity in sub-acute stroke patients’ wrists. They reported significant reductions in wrist flexor spasticity and increased activity in both wrist flexor and extensor muscles immediately and one-month post-intervention in the tDCS group compared to controls. Llorens et al. [41] examined the effects of combining tDCS with virtual reality (VR)-based therapy for chronic stroke patients. The result indicated that this approach significantly enhanced upper limb motor function, surpassing the outcomes of conventional physical therapy. Additionally, Kashoo et al. [42] investigated the benefits of combining tDCS with motor imagery (MI) and upper-limb motor training in chronic stroke rehabilitation. In conjunction with MI and functional training, they discovered that anodal tDCS stimulation applied to the affected M1 effectively reduced impairment and enhanced recovery in upper limb function. Furthermore, Ehsani et al. [43] applied anodal tDCS over the M1 of the ankle muscles with physical therapy. The group receiving the combined intervention showed improved EMG activity and more sustained clinical improvements. In addition, unilateral tDCS shows promise in enhancing physical therapy for lower limb recovery in stroke survivors. Seo et al. [44] observed enhanced walking function with anodal-tDCS and robotic-assisted gait training, with improvements in Functional Ambulatory Category (FAC) scores and the 6 min walk test four weeks post-treatment. Ehsani, Mortezanejad, Yosephi, Daniali, and Jaberzadeh [43] reported that anodal-tDCS reduced spasticity and improved muscle activity and balance, with lasting effects for a month. Qurat Ul et al. [45] showed that tDCS targeting the cerebellum or motor cortex, combined with virtual reality training, improved balance, gait, and cognition without significant differences between target areas. Interestingly, Duan et al. [46] demonstrated that even cathodal-tDCS with rehabilitation significantly improved lower limb function, as evidenced by FMA-LE scores and gait measures in subacute stroke patients.

Following a stroke, both the ipsilesional and contralesional hemispheres undergo significant changes. While the ipsilesional hemisphere’s alterations are directly linked to motor deficits, the contralesional hemisphere plays a more complex role in recovery. The contralesional hemisphere can support recovery by compensating for lost functions in the ipsilesional hemisphere [47]. However, its increased activity can also become maladaptive, hindering recovery by disrupting relearning processes in the damaged hemisphere [47,48,49]. Therefore, recent studies have modified their protocols by applying bi-hemispheric stimulation, aiming to regulate the imbalance between the hemispheres. In this approach, the anode is placed over the ipsilesional M1, and the cathode is positioned over the contralesional M1. Goodwill et al. [50] investigated the effects of a 3-week dual-tDCS combined with upper limb rehabilitation in chronic stroke survivors. The findings revealed that real-tDCS improved motor function and maintained these gains at a 3-week follow-up. Additionally, real-tDCS led to increased MEP amplitudes and enhanced corticospinal plasticity. Lefebvre et al. [51] reported that combining motor learning with dual-tDCS in stroke survivors increased functional brain connectivity, particularly in motor and premotor regions, suggesting improved cortical network efficiency. Kuo et al. [52] combined dual-tDCS with paretic hand exercise in subacute stroke survivors, and they reported that dual-tDCS successfully modulated ipsilesional M1 excitability and inter-hemispheric balance. Moreover, Garrido et al. [53] observed significant motor function improvements in acute and subacute stroke patients using dual-tDCS with constraint-induced movement therapy.

In addition, Andrade et al. [54] explored the impact of different tDCS montages on fall prevention and lower limb function in acute stroke patients, applying anodal, cathodal, bilateral, and sham tDCS across ten sessions over two weeks. The findings revealed that all active tDCS groups experienced reduced fall risks and improved lower limb function, with dual-tDCS stimulation showing the most significant benefits. Youssef et al. [55] compared the efficacy of dual-tDCS to anodal-tDCS in boosting motor function in sub-acute ischemic stroke survivors, finding substantial improvements in motor skills for both upper and lower limbs in both groups, with no discernible difference in effectiveness between the two tDCS approaches. Moreover, studies have also shown that combining dual-tDCS with physical therapy significantly enhances outcomes [56,57].

## 5. Transcranial Alternating Current Stimulation (tACS)

Brain activity exhibits rhythmic patterns that oscillate at specific frequencies. Unlike tDCS, which delivers a constant direct current, tACS applies weak sinusoidal currents at a fixed frequency, aiming to entrain the brain’s endogenous oscillations (Figure 1B). tACS does not significantly alter the overall rate of action potentials. Instead, it modulates the timing of neuronal spikes, resulting in a phase shift in endogenous oscillations, i.e., entrainment [58,59]. The modulation effect of tACS can also be explained through the concept of the Arnold tongue, which represents a triangular relationship between stimulation frequency and amplitude. This triangular area is centered at the frequency of the endogenous oscillation, illustrating how the effectiveness of tACS is influenced by the alignment of external stimulation parameters with the brain’s natural frequencies [58,60,61]. In addition, Ali, Sellers, and Frohlich [61] utilized large-scale simulations of cortical networks to investigate how tACS modifies these networks. They discovered that tACS entrainment can be mediated by the resonance dynamics of the brain. Liu, Voroslakos, Kronberg, Henin, Krause, Huang, Opitz, Mehta, Pack, Krekelberg, Berenyi, Parra, Melloni, Devinsky, and Buzsaki [3] also summarized that stochastic resonance, rhythm resonance, temporal biasing of neuronal spikes, entrainment of network patterns, and imposed patterns could affect the effect of tACS. In general, tACS’s widespread effects are attributed to two synergistic mechanisms: entrainment and neuroplasticity. Entrainment occurs when an external rhythm influences another system, causing it to synchronize its frequency and phase. Neuroplasticity, involving LTP/LTD processes, reinforces these online effects by either strengthening or weakening neural connections based on their activity levels [62,63].

As anticipated, tACS has demonstrated its capability of modulating cortical excitability when applied over M1 with various frequencies. Fresnoza et al. [64] observed that individual alpha frequency tACS increased MEP amplitudes post-stimulation in both young and old individuals, with a stronger effect in the young. The same group later also found that tACS improved old adults’ gross motor sequence scores [65]. Suzuki et al. [66] applied 10 Hz and 20 Hz tACS to the hand motor area for 20 min. Their findings revealed increased corresponding oscillatory activity at both frequencies in magnetic resonance images, demonstrating frequency-specific effects on motor cortex function. In addition, Guerra et al. [67] investigated the effects of beta and gamma tACS applied over M1 on repetitive finger tapping in healthy subjects. Their findings revealed that beta tACS decreased movement amplitude while gamma tACS increased it. However, other movement parameters and MEPs remained unchanged, suggesting a specific role for beta and gamma brain oscillations in the control of repetitive finger movements. Similarly, Miyaguchi et al. [68] explored the impact of beta and gamma tACS on motor performance by applying them to the M1 and cerebellar cortex in healthy adults. The study found no impact of beta-oscillation tACS on motor performance. However, gamma-tACS applied to M1 and the cerebellum significantly improved motor performance. Later, the same group investigated the impact of gamma-tACS on motor learning. They found that gamma-tACS significantly enhanced motor learning retention compared to sham stimulation. However, there were no differences in initial learning efficiency or the ability to re-learn between the gamma-tACS and sham groups [69]. Conflicting outcomes have also been reported. Geffen et al. [70] assessed the effects of slow oscillatory tACS (0.75 Hz) on motor cortex responsiveness in healthy subjects. Their results showed a significant increase in MEP amplitude following tACS. However, the study found no phase-dependent changes in excitability, suggesting that entrainment of endogenous neural oscillations might not be the primary mechanism underlying the observed effects. Pozdniakov et al. [71] reported that applying tACS at alpha and beta frequencies over M1 can increase cortical excitability during stimulation, especially at the beta frequency of 20 Hz. However, these excitability enhancements did not persist after the stimulation had ceased, indicating a lack of lasting offline effects. Therefore, applying tACS over the M1 region shows promise for modulating cortical activity, as demonstrated by changes in MEPs and cortical coherence. Importantly, different stimulation frequencies likely yield distinct modulation effects. However, direct evidence demonstrating that neural entrainment causes the observed changes in cortical excitability remains elusive.

Despite the extensive body of literature on applying tDCS to stroke survivors, the implementation of tACS on individuals with stroke remains considerably restricted. Chen et al. [72] investigated the effects of tACS at different frequencies on brain network integration and segregation in chronic stroke patients. The findings indicated that 20 Hz tACS might facilitate local segregation in motor-related regions and global integration at the whole-brain level. Naros and Gharabaghi [73] demonstrated that individualized tACS improved neurofeedback intervention accuracy in chronic stroke patients. Schuhmann et al. [74] found that high-definition tACS (HD-tACS) at alpha frequency effectively ameliorated hemi-spatial neglect symptoms in stroke patients by shifting attentional resources towards the contralesional hemifield. Wu et al. [75] reported significant neurological improvements in subacute stroke patients treated with tACS, as evidenced by reduced National Institutes of Health Stroke Scale scores. Later, Xie et al. [76] explored the benefits of 6 Hz tACS for chronic post-stroke aphasia, noting significant enhancements in various aspects of language performance, specifically in patients receiving active tACS targeted at the supplementary motor area.

## 6. Transcranial Random Stimulation (tRNS)

tRNS can be regarded as an adaptation of tACS, characterized by its delivery of stimulation across a wide frequency range instead of a single, fixed frequency (Figure 1C). Even if the exact physiological mechanisms of tRNS are not fully understood, following the mechanisms of tACS, former studies suggest that the modulation effects of tRNS may be enhanced by stochastic resonance and by repetitive activation of sodium channels that occur due to rectification when high-frequency stimulation is applied [77,78,79]. Interestingly, studies by Terney et al. [80] and Moret et al. [81] have found that the modulatory effects of tRNS are most pronounced within a wide-range high-frequency spectrum (100–700 Hz). This observation could be attributed to insufficient noise levels failing to adequately influence the activity of Na+ channels, thereby affecting their modulation [81]. In addition, Chaieb et al. [82] explored the effects of tRNS (101–640 Hz) on M1 cortical excitability and found that a 5 min application of tRNS led to a significant increase in excitability. Abe et al. [83] explored how tRNS (0.1–640 Hz) affects corticospinal excitability and motor performance. The study found that tRNS significantly increased MEP amplitudes and motor performance in healthy participants. Similarly, a recent review suggested that tRNS has the potential to increase motor cortex excitability, and this excitability enhancement is found to be dependent on the width of the frequency range used, the stimulation intensity, and duration [84].

In stroke motor recovery, a study investigating the combined effects of tRNS and upper limb training in stroke patients revealed that participants in the tRNS group exhibited significantly improved outcomes than the sham group [85]. Hayward et al. [86] explored whether tRNS applied over M1 can enhance upper limb recovery during reaching training in four stroke survivors with severe arm paresis. Participants underwent 12 training sessions, receiving either active or sham tRNS. They reported no adverse events and notable clinical improvements in motor outcomes in the active and sham groups. Moreover, Anwer, Waris, Gilani, Iqbal, Shaikh, Pujari, and Niazi [6] examined the combination of tRNS and functional electrical stimulation (FES) for improving upper extremity function in individuals with moderate-to-severe stroke for 18 sessions. Results showed significant improvements were observed in upper extremity impairment and function in the tRNS group, with no significant differences in motor function or grip strength between the groups.

## 7. Challenges in Transcranial Electrical Stimulation (tES)

Nevertheless, it is crucial to recognize that challenges in replication can arise, potentially impacting the reliability of conclusions drawn about the efficacy of tES techniques. In tDCS, Horvath et al. [87] pointed out that inter-subject variability, intra-subject reliability, challenges with sham stimulation and blinding, the impact of motor and cognitive activities on tDCS effects, and factors influencing electric current flow like hair thickness and electrode attachment methods should be carefully considered in the tDCS studies. In fact, a few recent studies have suggested that tDCS may not enhance cortical connectivity in healthy participants. Jonker et al. [88] investigated the impact of anodal tDCS applied over the M1 on cortical excitability in healthy participants, using MEPs as the measure. Despite previous findings suggesting that anodal tDCS can increase cortical excitability, this double-blind, placebo-controlled trial found no significant effect of tDCS on cortical excitability, nor did it find any interaction with individual-specific factors. Kudo et al. [89] investigated the effects of tDCS on corticomuscular coherence (CMC) and MEPs. CMC represents a measure of functional connectivity between cortical activity and muscular activity. However, their study found that tDCS did not significantly modulate either measure. Apsvalka et al. [90] investigated if anodal tDCS applied to M1 could enhance motor skill acquisition. The results indicated no significant benefit of active stimulation over sham in observing keypress sequences. Moreover, Gardi et al. [91] investigated the impact of tDCS device type and electrode size on cortical excitability. They reported that no significant differences were found in cortical excitability changes between different devices or electrode sizes, nor was there a significant effect of anodal tDCS alone.

Similar findings have also been reported in stroke rehabilitation concerning tDCS efficacy in augmenting stroke rehabilitation. In contrast to healthy individuals, the challenges of applying tDCS to stroke survivors can arise from the interhemispheric inhibition model and montage, optimal dose and safety concerns, interindividual variability, subject selection, outcome measures, or medication use [92]. Despite the positive outcomes from above, Rossi et al. [93] applied anodal tDCS to the affected M1 hemisphere in acute stroke patients. The motor deficits were evaluated using the Fugl-Meyer motor scale (FM) and the National Institute of Health Stroke Scale (NIHSS). The study found that both active and sham groups showed significant improvements in NIHSS and FM scores over time, but there was no significant difference in clinical outcomes between the anodal TDCS and sham groups. Similarly, Au-Yeung et al. [94] found that a 20 min session of cathodal tDCS applied to the contralesional M1 in chronic stroke survivors significantly improves hand dexterity. However, no significant hand dexterity improvements were observed with anodal tDCS targeting the lesioned hemisphere’s M1. Hamoudi et al. [95] explored the impact of anodal tDCS on motor skill learning in chronic stroke patients. They reported that while tDCS augmented motor skill learning during the online phase, these improvements were limited to the specific skills learned and did not generalize to broader motor functions.

Moreover, when combining tDCS with physical therapy in clinical practice, some studies have shown that tDCS does not augment the effectiveness of physical therapy. Straudi et al. [96] examined the effects of combining dual-tDCS with Robotic Assisted Training (RAT) for upper extremities in stroke survivors. Participants received either real or sham tDCS along with robotic therapy for 10 sessions. The results indicated that dual-tDCS might enhance the benefits of robotic therapy, but only when adjusted with stroke duration and type. Triccas et al. [97] explored the impact of anodal tDCS along with unilateral and three-dimensional RAT on the impaired upper limb in people with sub-acute and chronic stroke for 18 sessions. They found that the addition of tDCS showed no extra benefits, and RAT might be more advantageous in the sub-acute phase of stroke than the chronic phase. Moreover, Morone, Capone, Iosa, Cruciani, Paolucci, Martino Cinnera, Musumeci, Brunelli, Costa, Paolucci, and Di Lazzaro [10] examined dual-tDCS combined with exoskeleton RAT on upper limb motor functions in chronic stroke patients after 10 sessions of repetitive training. They reported that dual-tDCS combined with RAT did not further enhance recovery compared to controls. Bernal-Jimenez et al. [98] explored the effects of combining tDCS with RAT on the rehabilitation of upper limb function in chronic stroke patients for 20 sessions. They reported that the combination did not lead to significant improvements in the Fugl-Meyer Upper Limb Motor Score (mFM-UL), the Action Research Arm Test (ARAT), or the Functional Independence Measure (FIM) among the stroke patients. Moreover, two recent review articles [12,99] examined the effectiveness of integrating tDCS with RAT for upper limb function recovery after stroke. They concluded that while tDCS might enhance the effects of RAT on lower limb function, the combination does not appear to improve upper limb function, strength, spasticity, functional independence, or velocity of movement after stroke.

While the primary focus of this review is not on lower limb motor recovery, it’s pertinent to acknowledge that similar challenges have been observed regarding the effects of tDCS on lower limb recovery in stroke survivors. van Asseldonk and Boonstra [100] explored the impact of tDCS on walking in both healthy subjects and chronic stroke survivors, noting slight improvements in force production during walking among healthy participants with dual-tDCS, but no significant benefits for stroke survivors. Concurrently, Leon et al. [101] investigated the combination of tDCS with robotic gait training, finding no substantial difference in walking ability between those who received tDCS during training and those who underwent robotic training alone. Similarly, Kindred et al. [102] assessed the effects of high-definition tDCS (HD-tDCS) on gait and corticomotor response in post-stroke individuals, concluding that a single HD-tDCS session, regardless of being anodal or cathodal, failed to significantly alter gait kinematics, walking speed, or corticomotor responses. In addition, a research group conducted multiple studies on tDCS for lower-limb recovery in stroke survivors. Klomjai et al. [103] explored the effects of a single session of dual-tDCS combined with conventional physical therapy on lower limb function and gait, finding significant improvements in the Five-Times-Sit-To-Stand (FTSTS) test in the real tDCS group but no significant muscle strength changes. Subsequently, Klomjai et al. [104] assessed various tDCS setups over five days, noting that dual-tDCS offered the most significant lower limb motor function improvements. In contrast, Aneksan et al. [105] did not observe enhanced outcomes from five sessions of dual-tDCS with task-specific training. Similarly, Klomjai and Aneksan [106] found no significant lower limb performance improvements when dual-tDCS was applied during physical therapy. Additionally, recent reviews showed that tDCS had limited effects in an isolated treatment environment, but it is possible to improve lower limb functions when combined with other therapies [106,107,108,109].

Stimulation intensity and duration in tDCS significantly influence its modulation effects. However, there is no consensus on the optimal settings for either intensity or duration. Chew et al. [110] examined cortical excitability in healthy subjects with different anodal-tDCS intensities; significant MEP variations were observed between individuals across different current intensities, with 2 mA and 0.2 mA tDCS proving to be more effective in eliciting a clear response compared to 0.5 mA and 1 mA intensities. Additionally, notable variations were also seen within individuals across repeated sessions of identical tDCS. Vignaud et al. [111] compared the effects of tDCS’s duration (20 vs. 30 min) and intensity (1 vs. 2 mA) on cortical excitability. The findings revealed that a 20 min session of anodal-tDCS, irrespective of the intensity used, enhanced MEP responses. Conversely, a 30 min tDCS session did not alter cortical excitability. Esmaeilpour et al. [112] discussed whether increasing the electric current in tDCS improves its effectiveness under different models. However, their findings suggest a lack of clear understanding regarding the dose–response relationship in tDCS. Interestingly, another recent study explored individualized dose-control of tDCS to examine variability among healthy individuals. Their findings suggest that individualized dose-control of tDCS has the potential to reduce variance in cortical excitability [113]. In addition to the challenges mentioned above, the challenges of tDCS might be due to the complexity of motor skills, which involve both cortical and spinal and peripheral mechanisms. The task-specific effects of tDCS imply that its neuromodulation impact is closely associated with the neural circuits activated during specific training, indicating a lack of broad influence on other motor areas or unrelated skills [13,96,114]. These findings highlight the need for further studies to confirm these results and better understand the varying effects of tDCS in stroke rehabilitation.

However, due to the heterogeneity across studies, directly comparing the motor recovery effects between tDCS, tACS, and tRNS is challenging, as they do not adhere to the same stimulation paradigms or protocols or involve identical populations. To address the challenges of understanding the relative effectiveness of different tES techniques, several studies have endeavored to compare the efficacy of conventionally used tDCS with the more recently developed tACS and tRNS within the same population. In a study comparing the efficacy of tDCS, tACS, and tRNS on altering cortical excitability, each type of stimulation was applied over the M1 area in the same healthy adults at an intensity of 1.0 mA for 10 min on separate days. The findings revealed that tACS and tRNS led to an increase in MEPs compared to sham stimulation, while tDCS did not produce similar effects [115]. Krause et al. [116] investigated the effects of tACS and tDCS on motor sequence retrieval and reacquisition during early motor consolidation. Both tACS and tDCS showed facilitatory effects on motor sequence retrieval, with 20 Hz tACS being particularly effective in enhancing reaction times. Unfortunately, direct comparisons between tDCS, tACS, and tRNS in the motor cortex were quite limited. Although some comparisons did not specifically target the motor cortex, their findings merit consideration. Rohner et al. [117] aimed to directly compare the effects of theta-tACS and anodal tDCS on working memory (WM) performance. Their results revealed that tACS resulted in a greater improvement in reaction time for correct hits than tDCS. Moreover, Kim et al. [118] explored the efficacy of tACS and tDCS in enhancing cognitive function in patients with mild cognitive impairment. Participants received both gamma-tACS (40 Hz) and tDCS at the same intensity applied to the dorsolateral prefrontal cortex. The study found that gamma-tACS improved cognitive performance compared to tDCS and sham treatments. In contrast, tDCS did not demonstrate significant differences from sham in any of the cognitive test scores. In addition, a recent review by Senkowski et al. [119] compared the effects of tDCS and tACS on working memory (WM) in healthy adults, drawing from 43 studies. Results indicated a limited impact of single-session tDCS on WM, while tACS demonstrated frequency-dependent effects, particularly with frontoparietal stimulation. However, to the best of my knowledge, no study has directly compared the effectiveness of tDCS, tACS, and tRNS in stroke motor recovery.

## 8. Advantages of Using tACS/tRNS in Cortical Excitability Modulation and Motor Recovery

After a stroke, the brain’s neural oscillation patterns change based on lesion location and severity. Alpha waves, known for their role in relaxation and information processing, slow down and become more synchronized. Conversely, beta waves, associated with motor control, exhibit increased activity in both hemispheres. Additionally, gamma waves, crucial for sensory integration and information binding, experience disruption [120]. However, brain oscillations begin to show different characteristics associated with improved outcomes in the chronic stroke recovery phase. Studies have shown that a decrease in the synchronization of alpha waves is linked to better motor function [121]. Furthermore, increased coherence between beta waves in the motor cortex and other brain regions during the acute phase has been associated with improved functionality later [122]. Interestingly, the role of beta waves appears to be hemisphere-specific, with higher power in the affected hemisphere correlating with better motor recovery, while the opposite is true for the unaffected hemisphere. Finally, an increase in gamma wave power in the affected hemisphere emerges as a promising target for stroke rehabilitation, as it has been linked to positive outcomes [120,121,123].

To address why tACS/tRNS might have potential in stroke motor recovery, it is reasonable to have the hypothesis that the entrainment of neurons could achieve better performance than simply depolarization or hyperpolarization of neurons. In contrast to tDCS, Wischnewski et al. [124] reported in a review that beta-tACS significantly increases M1 excitability. A notable finding was that tACS intensities above 1 mA peak-to-peak robustly increased M1 excitability. A potential advantage of tACS lies in the selection of stimulation frequency, aimed at modulating task-relevant physiological processes. In contrast to tDCS, whose effects are primarily contingent upon electrode placement and current intensity, tACS introduces an additional dimension through the manipulation of the stimulation frequency [119]. The effects of stroke on neural oscillations depend on the damage’s severity and location. Stroke survivors typically experience a reduction in low-frequency wave power, with alpha oscillations showing decreased frequency and increased synchronization. On the other hand, beta oscillation power usually increases across both hemispheres [125]. Therefore, simply depolarizing and hyperpolarizing the motor cortex might not precisely address the changes in neural oscillations, suggesting that tDCS may not effectively modify neural oscillations in a frequency-specific way.

Moreover, unlike fixed-frequency protocols, tACS can be adjusted to match an individual’s endogenous frequency. Fresnoza, Christova, Feil, Gallasch, Korner, Zimmer, and Ischebeck [64] applied individual alpha frequency tACS to the motor cortex in both young and older groups, observing increased cortical excitability post-stimulation in both. Similarly, Schilberg et al. [126] demonstrated that tACS set to individual beta band frequencies can modulate MEPs. Therefore, tailoring tACS to individual frequencies may enhance its effectiveness, potentially aligning more closely with the brain’s intrinsic neuronal oscillations. This suggests that individualized tACS protocols could play a significant role in stroke patient motor recovery in the future. Finally, the modulation effect of tACS can be state-dependent. Alagapan et al. [127] applied tACS across different behavioral states: eyes open, eyes closed, and during a task. They found that the effect of tACS was dependent on the behavioral state. The complexity and dependency of brain activity upon the current behavioral state demonstrate the strength of tACS to accommodate more variable applications over the limitations of other tools. Therefore, tACS/tRNS may be a good tool to augment the intervention outcomes.

## 9. Limitations

The current review was primarily focused on upper-limb motor recovery. The neural oscillation patterns associated with lower limb movements (such as walking) or balance control might differ significantly from those of the upper limb, potentially complicating the interpretation of tES modulation effects. However, future reviews should specifically address lower limb recovery in stroke, exploring how various tES techniques influence motor functions in this area. In addition, although there is a growing body of research on tACS in motor recovery, the existing literature on both tACS and tRNS remains too scarce to draw definitive conclusions about their modulatory effects on stroke survivors.

## 10. Conclusions

In this review, we primarily focused on studies published within the past 10 years examining tES modulation of healthy motor cortical excitability and stroke upper limb motor recovery. The field of tES modulation has gained tremendous attention, as evidenced by the increasing number of publications. However, despite its emergence as a promising technique with advantages for research and clinical settings, replicating the benefits of tES remains challenging due to variability in study designs, participant characteristics, and stimulation protocols. While tDCS is the most frequently used tES technique in stroke motor recovery, its efficacy in augmenting the effects of physical therapy remains uncertain. In contrast, emerging tES techniques like tACS and tRNS, with distinct mechanisms from tDCS, show potential in preliminary stroke motor recovery studies. The complexity of neural networks suggests that more sophisticated approaches capable of targeting specific neural oscillations may offer an alternative for stroke motor rehabilitation and enhance the effects of physical therapy. Therefore, future progress hinges on understanding neural mechanisms and refining tES techniques for consistent, therapeutically valuable results. As the field develops, modified tES holds the potential to become a powerful neuromodulatory tool, enhancing stroke upper limb motor rehabilitation.

## Data Availability

This narrative review article synthesizes and analyzes findings from existing literature. No new data were generated for this study. All referenced materials and sources are publicly available and can be accessed through PubMed. Since this work involved the aggregation and interpretation of existing public data, no specific datasets were created.
